# Double‐wall sign because of intestinal perforation

**DOI:** 10.1002/jgf2.508

**Published:** 2021-11-02

**Authors:** Toshimasa Yamaguchi

**Affiliations:** ^1^ Primary Care and Advanced Triage Section Osaka City General Hospital Osaka Japan

**Keywords:** double‐wall sign, duodenal ulcer, perforated peptic ulcer, pneumoperitoneum

## Abstract

This study sought to describe the case of an 86‐year‐old man who presented to our hospital complaining of abdominal pain, abdominal distention, and loss of appetite for 4 days prior. This case suggests that an amount of accumulated air clearly highlights the intestinal wall, like a “double‐wall sign,” even when the patient is standing.

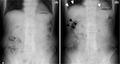

An 86‐year‐old man who had prostate cancer, bladder cancer, type 2 diabetes mellitus, and a history of duodenal ulcer presented at our hospital with abdominal pain, abdominal distention, and loss of appetite for 4 days prior. Physical examination revealed mild tenderness, with no rebound tenderness, on the right upper abdomen, and attenuation of bowel sounds. Abnormalities were not detected during the initial plain erect abdominal radiograph (Figure 1A). Therefore, the patient was treated conservatively and followed up, with a diagnosis of abdominal symptoms of unknown cause. However, he used an enema at home and presented to the hospital again 2 days later, complaining of exacerbated abdominal pain. On palpation, tenderness was noted across the upper abdomen. Another abdominal radiograph was obtained (Figure 1B) and compared with the preceding radiograph, which had already disclosed air on both sides of the wall of the transverse colon, indicating pneumoperitoneum; however, it had not been detected during the first presentation. Bilateral subdiaphragmatic free air was additionally revealed in the second radiograph. Thus, we assumed that the intestinal perforation had occurred before the preceding visit, and increasing intestinal intraluminal pressure because of the enema had led to the worsening of his condition. Subsequent plain computed tomography revealed free air surrounding the duodenal bulb and the liver surface. An emergency laparotomy was performed, and an ulcerated duodenal bulb wall perforation was revealed. The patient underwent simple perforation closure, and the postoperative course was uneventful.

**FIGURE 1:**
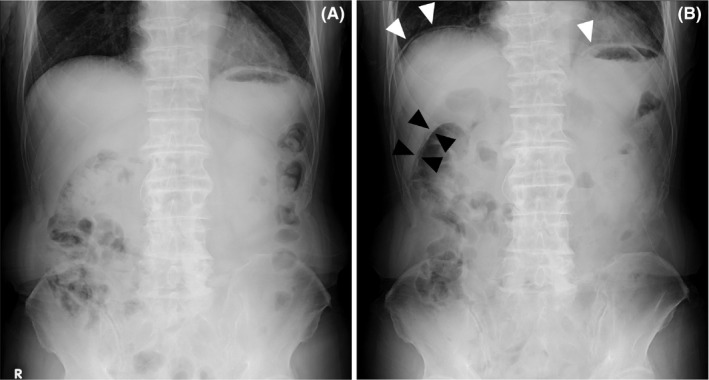
(A) Abdominal radiograph at the first presentation. (B) Abdominal radiograph demonstrating a “double‐wall sign” in the transverse colon in the right subhepatic space (black arrowheads), which could not have been detected in the preceding radiograph (A) during the first presentation, and additional bilateral subdiaphragmatic free air 2 days after the first presentation (white arrowheads)

Plain abdominal radiographs are helpful for diagnosing intestinal perforation if free air is observed. A double‐wall sign, also known as the Rigler sign, indicates the presence of air along the luminal and peritoneal cavities outlining both sides of the intestinal wall in the supine position. It is one of the most useful findings for detecting pneumoperitoneum with plain radiographs.^1^ Free air from the duodenal bulb perforation tends to accumulate in the anterior right subhepatic space because of anatomical features. In this case, it was suggested that such accumulated air clearly highlighted the intestinal wall, like a “double‐wall sign,” even in the standing position. A chest radiograph is often performed to disclose the presence of subdiaphragmatic free air suggesting intestinal perforation and intrathoracic diseases. Therefore, a supine abdominal radiograph is occasionally combined with a chest radiograph in the acute abdomen, and an erect abdominal radiograph is not frequently routinely performed. However, gastric and duodenal ulcers account for a large proportion of the causes of acute abdomen with upper abdominal pain.^2^ Furthermore, it has been reported that the mortality rate of a perforated peptic ulcer is significantly higher in patients older than 70 years.^3^ The radiographic finding of an anterior right subhepatic “double‐wall sign” could be regarded as an initial sign of pneumoperitoneum owing to perforated duodenal ulcer before the appearance of subdiaphragmatic free air. It is important to select radiographic procedures according to the patient's condition, especially with acute abdomen in the elderly.

## CONFLICT OF INTEREST

The authors have stated explicitly that there are no conflicts of interest in connection with this article.

## CONSENT FOR PUBLICATION

Patient consent has been obtained.
